# 

**Published:** 1881-10

**Authors:** 


Meetings of Societies in October.—American Academy of Dental Science at Boston, Mass., 26th; Fifth and Sixth District Dental Societies of the State of New York, at Courtland, N.Y., 6th.

WILL *YOU* PLEASE SEND IN *YOUR* SUBSCRIPTION MONEY *NOW*?

Money is at OUR RISK ONLY when sent by Post Office Money Order, Registered Letter, or Check on Banks in New York City, made payable to INDEPENDENT PRACTITIONER, 20 Courtlandt street, N. Y. City.

If you prefer to send check on your private bank, unless located in the city

